# Optimized size exclusion chromatography demonstrates that extracellular vesicles are the key RNA carriers of ALK translocations in non-small cell lung cancer cell line secretome and patient plasma

**DOI:** 10.20517/evcna.2025.14

**Published:** 2025-06-18

**Authors:** Beatriz Benayas, Estela Sánchez-Herrero, Lucía Robado de Lope, Joaquín Morales, Soraya López-Martín, Mariano Provencio, Mar Valés-Gómez, Atocha Romero, María Yáñez-Mó

**Affiliations:** ^1^Centro de Biología Molecular Severo Ochoa (CBM), IIS-Princesa, Universidad Autónoma de Madrid, IUBM, Madrid 28049, Spain.; ^2^Immunology and Oncology Department, Spanish National Centre for Biotechnology (CNB-CSIC), Madrid 28049, Spain.; ^3^Liquid Biopsy Laboratory, Medical Oncology Department, Hospital Puerta de Hierro, IDHIPISA, Madrid 28022, Spain.; ^#^Authors contributed equally.

**Keywords:** Non-small-cell lung cancer, extracellular vesicles, ALK, size-exclusion chromatography, liquid biopsy

## Abstract

**Aim:** Identification of *ALK* fusions in non-small cell lung cancer (NSCLC) is key to determining eligibility for treatment with ALK inhibitors that markedly improve patients’ quality of life and survival outcomes. Circulating RNA, associated with various carriers including extracellular vesicles (EVs), lipoproteins (LPPs), or protein complexes, presents a viable target for the identification of ALK fusions by liquid biopsy. Our aim was to characterize the specific carrier of *ALK* fusion RNA, a crucial step in the development of diagnostic methods for clinical use.

**Methods:** We employed optimized size-exclusion chromatography (SEC) to separate EVs, LPPs, and protein-enriched fractions from *ALK*-positive NSCLC cell lines and from pools of plasma obtained from NSCLC patients with *ALK* translocations. We optimized RNA fusion transcript detection using digital PCR (dPCR).

**Results:** Protein analyses confirmed the successful resolution of EVs, LPPs, and protein fractions by optimized SEC. Our dPCR results indicated that *ALK* fusions were more prevalent in tetraspanin-enriched SEC fractions from NSCLC cell lines, suggesting that EVs serve as the primary carrier for *ALK* fusion RNA. After optimization for larger volumes of samples of the RNA isolation protocol, we could also demonstrate that *ALK* fusion transcripts were found exclusively in EVs from patient plasma. Of note, the circulating number of copies of the transcript was below 5 copies/mL.

**Discussion:** Our findings underscore the potential of EV-associated RNA as a promising source for detecting *ALK* fusion variants in plasma samples from NSCLC patients, offering a non-invasive diagnostic approach with significant clinical implications.

## INTRODUCTION

Lung cancer is the second most diagnosed cancer type (2.2 million new cases in 2020) and the leading cause of cancer-related deaths worldwide (1.8 million deaths in 2020). Non-small cell lung cancer (NSCLC) is the most common type of lung cancer, accounting for 85% of cases^[1]^. In 20%-40% of NSCLC patients, a genetic alteration is detected, which can identify patients who may benefit from approved targeted therapies^[2]^. Thus, a multigene evaluation for NSCLC tumors is recommended according to clinical guidelines^[3]^, particularly the study of the *ALK* (anaplastic lymphoma kinase), *EGFR*, and *ROS1* genes. However, heterogeneity in the anatomy of the tumor often makes it challenging to get access to enough material for biomarker assay.

The *ALK* gene encodes a transmembrane receptor of the tyrosine kinase type. Rearrangements in this gene occur in 3%-7% of NSCLC patients, leading to the oncogenic activation of this receptor^[4,5,6]^. Currently, there are multiple tyrosine kinase inhibitors against ALK-positive NSCLC, and their use has demonstrated significant improvements over chemotherapy in patient quality of life and survival outcomes. Thus, early identification of these patients would provide a tremendous opportunity to optimize their treatment^[7,8]^.

Liquid biopsy enables the collection of information about a patient’s condition through a minimally invasive, simple, and rapid procedure based on the analysis of biological fluids. Liquid biopsy can provide more comprehensive information than tissue biopsy, since tumor heterogeneity can result in biopsy samples not completely representative of the tumor^[9]^. In contrast, genetic analyses of liquid biopsy samples enable analysis of all the different components that tumors release into the bloodstream: circulating free DNA (cfDNA), circulating free RNA (cfRNA), circulating tumor cells (CTCs), tumor-educated platelets, and extracellular vesicles (EVs)^[10,11]^.

cfDNA is released by any normal or tumor cell (circulating tumor DNA: ctDNA) into the bloodstream following apoptosis or necrosis processes and is the most commonly analyzed component for the detection of genetic alterations in cancer^[12]^. In contrast, RNases present in the bloodstream rapidly degrade cfRNA after its release, limiting its suitability for diagnostic purposes. However, certain types of RNAs in the blood can be stabilized by associating with various carriers, including subpopulations of lipoproteins (LPPs) such as HDL^[13]^, VLDL^[14]^, or LDL^[15]^, as well as protein complexes containing Argonaute 2^[16]^. Additionally, EVs are secreted by every cell and play a crucial role in intercellular communication by transporting and preserving the integrity of lipids, proteins, and nucleic acids in both physiological and pathological situations^[17]^. The detection of proteins, nucleic acids, and even specific metabolites present in tumor-derived EVs has been shown to enable differentiation between patients and healthy individuals, between patients in early or advanced stages, to predict or study treatment response, to assist with disease prognosis, and to identify patients with metastasis^[18]^.

Detection of *ALK* fusion using cfDNA as the starting material is challenging due to the unknown genomic breakpoints, the potential involvement of large genomic regions, and the fragmentation of cfDNA, which hampers detection. In contrast, detecting translocations at the RNA level is simpler because the fusion sequences are typically known. However, since cfRNA is rapidly degraded, it is preferable to focus on RNA species associated with stable RNA carriers present in plasma. Identifying the most relevant RNA carrier is critical for developing clinically applicable methods to isolate plasma components that enable reliable ALK fusion testing in NSCLC patients, minimizing analytical errors (false negatives/positives).

Although previous studies have reported the detection of translocation RNA in plasma using various strategies (ExoEasy columns^[19]^ or differential ultracentrifugation^[20]^), these methods tend to co-isolate EVs with LPPs^[21]^ or protein aggregates^[22]^. Therefore, the aim of this study was to identify which plasma components carry the EML4-ALK translocation transcript. To achieve this, we fractionated both the culture media conditioned by NSCLC cell lines and patient plasma samples using an optimized size-exclusion chromatography (SEC)^[23]^ protocol that effectively separates EVs, LPPs, and soluble proteins.

## METHODS

### Cells

The lung adenocarcinoma cell lines H3122 and H2228 were cultured in RPMI (Sigma Aldrich, R6504, batch 1003327517) supplemented with 10% FBS (Sigma Aldrich, F7524, batch 1660391), L-glutamine (Thermo scientific, G0063, batch DDNQO-JA) (1 mM), sodium pyruvate (Merck Millipore, 1.066.190.250, batch K47833519645) (1 mM), non-essential amino acids (Merck Millipore, 1010070100, and Sigma Aldrich A4284, A7219, G8415, P5607) (0.1 mM), HEPES (Sigma Aldrich, H3375, batchSLBS2995V) (10 mM), penicillin (Thermo scientific, J63901.22 batch U09K047) (100 U/mL), and streptomycin (PanReac AppliChem, A1852,0025, batch 4J020420) (100 µg/mL). All cells were grown at 37 °C in a humidified atmosphere with 5% CO_2_.

For EV production, a specific complete medium was used, supplemented with 5% FBS previously depleted of EVs, obtained by ultracentrifugation at 120,000 × g for 16 h. H3122 cells were seeded at a density of 2 × 10^6^ cells in p150 plates and cultured for 5 days until reaching confluence (approximately 18 × 10^6^ cells per plate). For the H2228 cell line, 2.75 × 10^6^ cells were seeded in p150 plates; once adhered on the next day, the medium was changed to fresh medium for EV production, and cells were grown for 3 days until doubling in number (approximately 5.5 × 10^6^ cells per p150).

### Plasma samples

Blood samples were collected from two NSCLC patients (with *EML4-ALK* translocation variant 3) at Hospital Universitario Puerta de Hierro-Majadahonda. Both patients were diagnosed with histologically confirmed ALK-positive NSCLC and presented with metastatic disease (Stage IV), according to the 8th edition of the International Association for the Study of Lung Cancer (IASLC) Staging System. Plasma samples were collected at baseline (before treatment initiation). The study protocol was approved by the Hospital Universitario Puerta de Hierro Ethics Committee (internal code PI 118/22). Prior to enrolment, appropriate written informed consent was obtained from all patients. Blood samples were collected in 10 mL “Cell-Free DNA BCT®” tubes (Streck) and centrifuged at 2,000 × g for 10 min at room temperature (RT) to obtain plasma. After obtaining the plasma, cellular debris was removed by centrifugation at 3,000 × g for 20 min at RT, and the plasma was frozen at -80 °C until use. Again, before being used, the samples were thawed at 4 °C, and any remaining aggregates were removed by centrifuging at 2,500 × g for 15 min at RT. The SPREC code for the samples would be PL2-ZZZ-A-A-D-N-A^[24]^.

### Isolation of EVs

Cell-conditioned medium (CM) was centrifuged for 5 min at 500 × g to remove remaining cells and then for 30 min at 3,200 × g at RT to eliminate cellular debris and apoptotic bodies. The CM from two p150 plates (≈ 32 mL) of H3122, or four p150 plates (≈ 64 mL) of H2228, free from cellular debris, was concentrated using Amicon Ultra-15 filters (100K, Merck Millipore, UFC910024, batch 251300) to a final volume of ≈ 500 µL.

For SEC, empty columns with cellulose top and bottom filters were used. The columns were packed up to 10 mL (for cell CM) or 13 mL (for plasma) with a previously optimized resin: 4% Rapid Run Agarose Bead Fine from Agarose Beads Technologies (ABT, 4RRF, batch H-08616F-4RR01)^[23]^, and equilibrated with 2 column volumes of filtered PBS.

The concentrated CM (≈ 500 µL) was added to the column, and 25 fractions of 500 µL each were collected by gravity elution. For plasma, 8 mL of pooled plasma from the two NSCLC patients carrying variant 3 of EML4-ALK translocation were fractioned using 8 columns (1 mL/column) and 30 fractions of 500 µL each were collected during the sample elution using filtered PBS as the elution buffer. PBS was filtered with 0.22 m pore diameter filters from Merck Millipore (SLGS033SS, batch 307215)

### Nanoparticle tracking analysis and dynamic light scattering

EV fractions were analyzed by nanoparticle tracking analysis (NTA) using a NanoSight NS300 (Malvern Instruments Ltd.) equipped with a 532 nm laser. Prior to analysis, samples were diluted 100-fold in filtered PBS. Using NTA 3.0 software, five videos of 60 s each were analyzed for each sample, with the camera level set to 11 and the analysis threshold set to 5.

EV and LPP fractions were analyzed by dynamic light scattering (DLS) using a Zetasizer Nano ZS (Malvern Panalytical). Samples were diluted 500-fold in filtered PBS and three independent measures/sample were obtained.

### Transmission electron microscopy

Isolated EVs were diluted 1:2 (EVs derived from CM) or 1:4 (EVs derived from plasma) in filtered PBS, absorbed onto carbon-coated nickel grids, and contrasted with 2% uranyl acetate (negative staining). Sample visualization and image acquisition were performed on a JEM1400 Flash transmission electron microscope (Jeol).

### Dot blot and western blot

For dot blot analyses of SEC samples, 1 µL of each fraction was directly deposited onto a nitrocellulose membrane (GE Healthcare) and allowed to dry.

For Western blot analyses of SEC-fractionated plasma, 20 µL of each fraction were loaded onto SDS PAGE polyacrylamide gels. Samples were lysed with non-reducing Laemmli buffer and boiled for 5 min at 96 °C. Following electrophoresis, proteins were electro-transferred to PVDF membranes using a Transfer-Blot Turbo system (Bio-Rad).

Dot blot or Western blot membranes were blocked with 5% skim milk in TBS/0.1% Tween-20 for 30 min. They were then incubated with the specified primary antibodies for 1 h at RT or overnight at 4 °C. For EV detection, primary antibodies against tetraspanins (hybridoma supernatant) CD9 (clon VJ/1.20), CD81 (clon 5A6; provided by Dr. S Levy, Stanford, USA)^[25]^, and CD63 (clon Tea3.10)^[26]^ were employed. For LPP detection, we used antibodies against Apolipoprotein B (Calbiochem, 178467) and Apolipoprotein E (Calbiochem, 178479).

After washing, the membranes were incubated with HRP-conjugated secondary antibodies for 30 min at RT. Membranes were developed with Super Signal West Femto HRP substrate (Thermo Scientific, 34096, batch UC280371). Images were acquired using a LAS 4000 mini system (General Electrics) and processed with Fiji ImageJ.

### Protein semi-quantification

The protein elution profile after SEC was obtained by measuring the absorbance of each fraction at 280 nm using a Nanodrop One (Thermo Scientific).

### RNA extraction, quantification and quality assessment

SEC fractions enriched in either EVs, LPPs, or free proteins were pooled and used for RNA extraction using the Norgen Plasma/Serum Exosome and Free-Circulating RNA Isolation kit (59600; batch 601791), or a TRIzol/chloroform extraction method followed by RNA binding to Norgen kit columns or Qiagen RNeasy MinElute Spin Columns (exoRNeasy Midi Kit, 77144, batch: 175011860).

The Norgen Plasma/Serum Exosome and free-circulating RNA Isolation kit was used following the manufacturer’s instructions. The volume of the reagents was adapted to sample volume [1,125 µL of lysis buffer A, 140.64 µL of lysis enhancer B, and 2.55 mL of ethanol (96%) per 1.5 mL of sample] and RNA was eluted in 40 µL of elution buffer.

TRIzol/chloroform lysis was also performed for high-volume samples (> 1.5 mL). Briefly, TRIzol (LS REAGENT, 10296010, batch 98500402) was added to the samples (ratio 3:1), mixed, and incubated for 15 min at RT. Chloroform (194002, batch:SR05772) was then added (ratio 1:5 TRIzol) and samples were centrifuged for phase separation. After the recovery of the aqueous phase, samples were mixed with 96%-100% ethanol and further purification was performed using the Norgen Mini Spin Columns or the Qiagen RNeasy MinElute Spin Columns following the manufacturer’s instructions.

RNA concentration was measured using the Qubit^TM^ RNA HS Assay kit (Invitrogen, Q32855, batch 2581673) on a Qubit4 fluorometer, and the RNA profile was analyzed with the Agilent RNA 6000 Pico Kit (5067-1513, batch: Agilent RNA 6000 Pico Reagents 2240, RNA Pico Chips BN10BK30) using Agilent 2100 Bioanalyzer (Agilent Technologies).

### Reverse transcription and digital PCR analysis

Reverse transcription of 6.5 µL of isolated RNA was performed using the PrimeScript RT Reagent kit (Takara, RR037A, batch: AM51563A) following the manufacturer’s instructions.

The presence of *EML4-ALK* translocation was checked by digital PCR (dPCR) using the Digital QuantStudio® 3D Digital PCR 20K chip (A26316, batch 2310353). The dPCR reaction mixture contained cDNA, QuantStudio 3D Master Mix, and TaqMan oligo for target detection. *PUM1* (Pumilio RNA Binding Family Member 1, dPCR TaqMan® Assay ID: Hs00472881_m1, Dye VIC-MGB, batch p120602-007 e07) was used as the endogenous control. For translocation detection, either TaqMan oligos EML4 (13):ALK (20) (Hs03654556_ft, Dye FAM-MGB, batch 1611348) or TaqMan EML4 (6a/b):ALK (20) (Hs03654558_ft, Dye FAM-MGB, batch 1597341) was included to for variants 1 and 3, respectively. 6.5 µL of cDNA was used for a final volume of 15 µL, of which 14.5 µL was loaded onto a QuantStudio 3D Digital PCR 20K chip by QuantStudioTM 3D dPCR Chip Loader (Applied Biosystems). Each dPCR included a blank (no cDNA), and a positive control (cell lysate from H2228 or H3122) [Supplementary Figure 1]. With these controls, thresholds of positive signals are determined and applied to negative and ALK-carrying plasma samples. The dPCR program started with an initial denaturation at 96 °C for 10 min, followed by 45 cycles of 2 min at 56 °C and 30 s at 98 °C, then a step of 10 min at 72 °C, and finally, the samples were left for at least 30 min at 22 °C.

After the dPCR reaction, the chips were read twice, and the results were analyzed using the QuantStudio® 3D AnalysisSuite^TM^ Cloud program. The mutant allele frequency (MAF) was calculated as the ratio of mutant molecules to the total number of molecules.

## RESULTS

### Detection of *EML4-ALK* translocation in conditioned medium of NSCLC cell lines

Since cfRNA is highly unstable in biological fluids, it is important to consider RNA carriers that protect it from external degradation as potential sources of genetic material for the detection of *EML4-ALK* translocation via liquid biopsy. In previous reports where translocation RNA was detected in EVs, the isolation methodology employed could not exclude the possibility that the transcript may also be transported by other carriers, such as LPPs or protein aggregates. To overcome these limitations, we employed SEC with an optimized resin^[23]^ that enables the separate analysis of EVs, LPPs, and protein fractions in the secretome.

**Figure 1:**
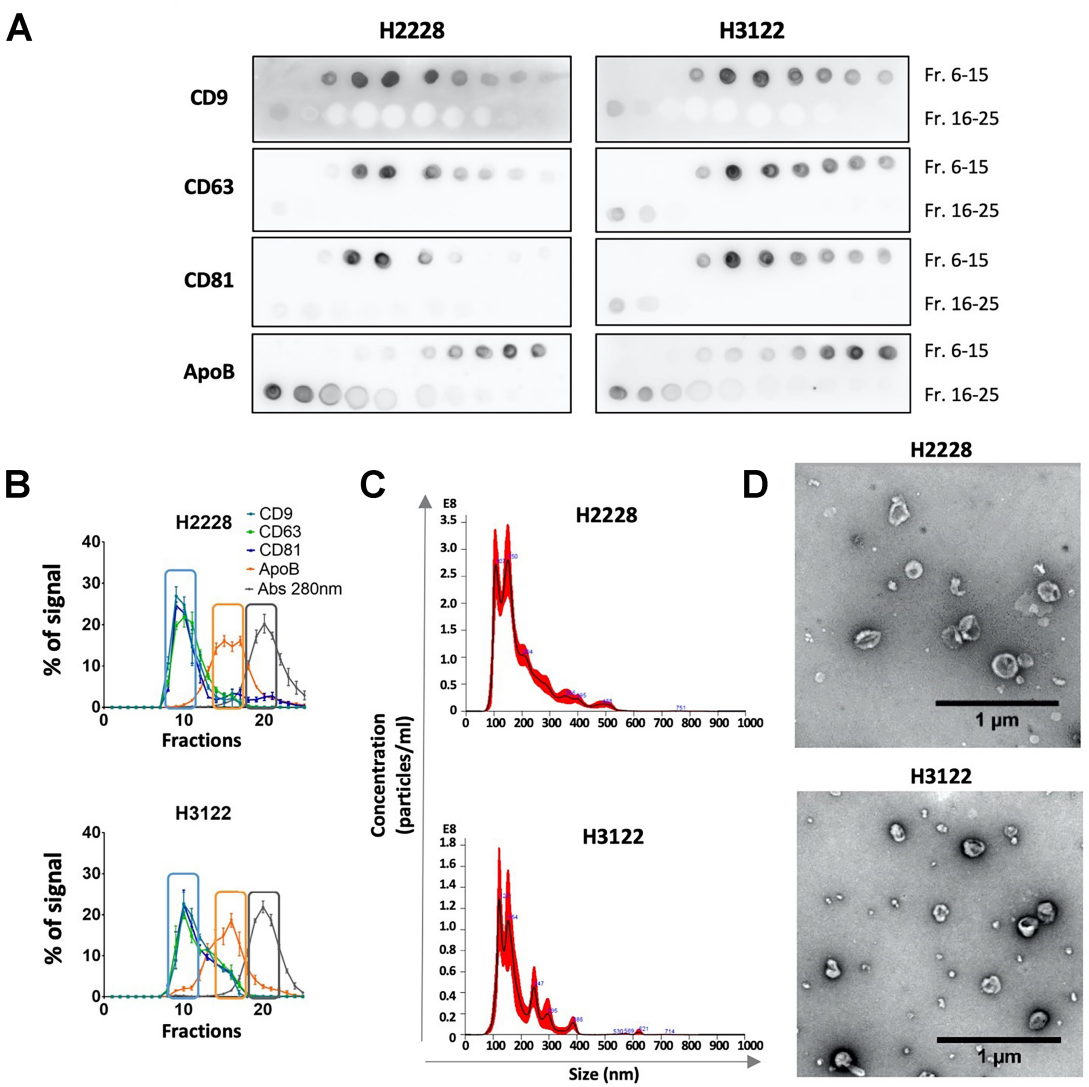
Separation of the three main components of conditioned media (EVs, LPPs, and protein) by optimized SEC. (A) Representative dot blots of EV markers (CD9, CD63, and CD81) and the LPP marker (ApoB) across SEC fractions. Graphing software: LAS 4000 mini system and Fiji ImageJ; (B) Elution profiles of EVs, LPPs, and soluble proteins. Densitometry analysis of dot blots for EV markers (CD9, CD63, and CD81) and the LPP marker (ApoB), along with absorbance at 280 nm for total protein, are shown as the % of the total signal for each marker (normalized to 100% as the sum of signals across all eluted fractions). Squares indicate the fractions pooled for further analysis of EVs, LPPs, and protein. Data represent mean ± SEM from at least three independent experiments. Graphing software: GraphPad Prism; (C) Size distribution of particles in EV-enriched fractions, analyzed by NTA. Graphing software: NTA 3.0 software (stated under methods); (D) Representative TEM images of negatively stained EV-enriched fractions. Scale bars = 1 μm. Graphing software: JEM1400 Flash transmission electron microscope (Jeol) and Image J. EVs: Extracellular vesicle; Fr.: fraction; LPP: lipoprotein particle; SEC: size-exclusion chromatography; NTA: nanoparticle tracking analysis; TEM: transmission electron microscopy; SEM: standard error of the mean.

First, we analyzed CM from NSCLC cell lines H3122 and H2228, which respectively carry variants 1 and 3 of the *EML4-ALK* translocation. The main components of the CM were efficiently separated by SEC using an optimized resin, as revealed in the elution profile of EVs [determined and quantified by dot blot [Figure 1A and B] with antibodies against different tetraspanins (CD9, CD81, and CD63)], LPPs (with anti-ApoB), and soluble protein, determined by measuring the absorbance at 280 nm of the eluted fractions [Figure 1B].

The three fractions most enriched in each of the main secretome components (EVs, LPPs, and proteins) were pooled (typically fractions 9-11 for EVs, 15-17 for LPPs, and 19-21 for soluble proteins). EV-enriched pools were analyzed by NTA [Figure 1C] and transmission electron microscopy (TEM) [Figure 1D] following MISEV guidelines^[27]^. The zeta potential of both EV and LPP fractions was measured by DLS using a 1:500 dilution of the samples in PBS at neutral pH. In both cases, the zeta potential of EV samples (-8.3 ± 1.27 mV for H3122 and -11.77 ± 0.45 mV for H2228) was clearly more negative than that of the LPP samples (-4.8 ± 0.6 mV for H3122 conditioned media and -5.32 ± 0.14 mV for H2228 conditioned media), confirming the purity of the isolated EV fractions.

**Figure 2:**
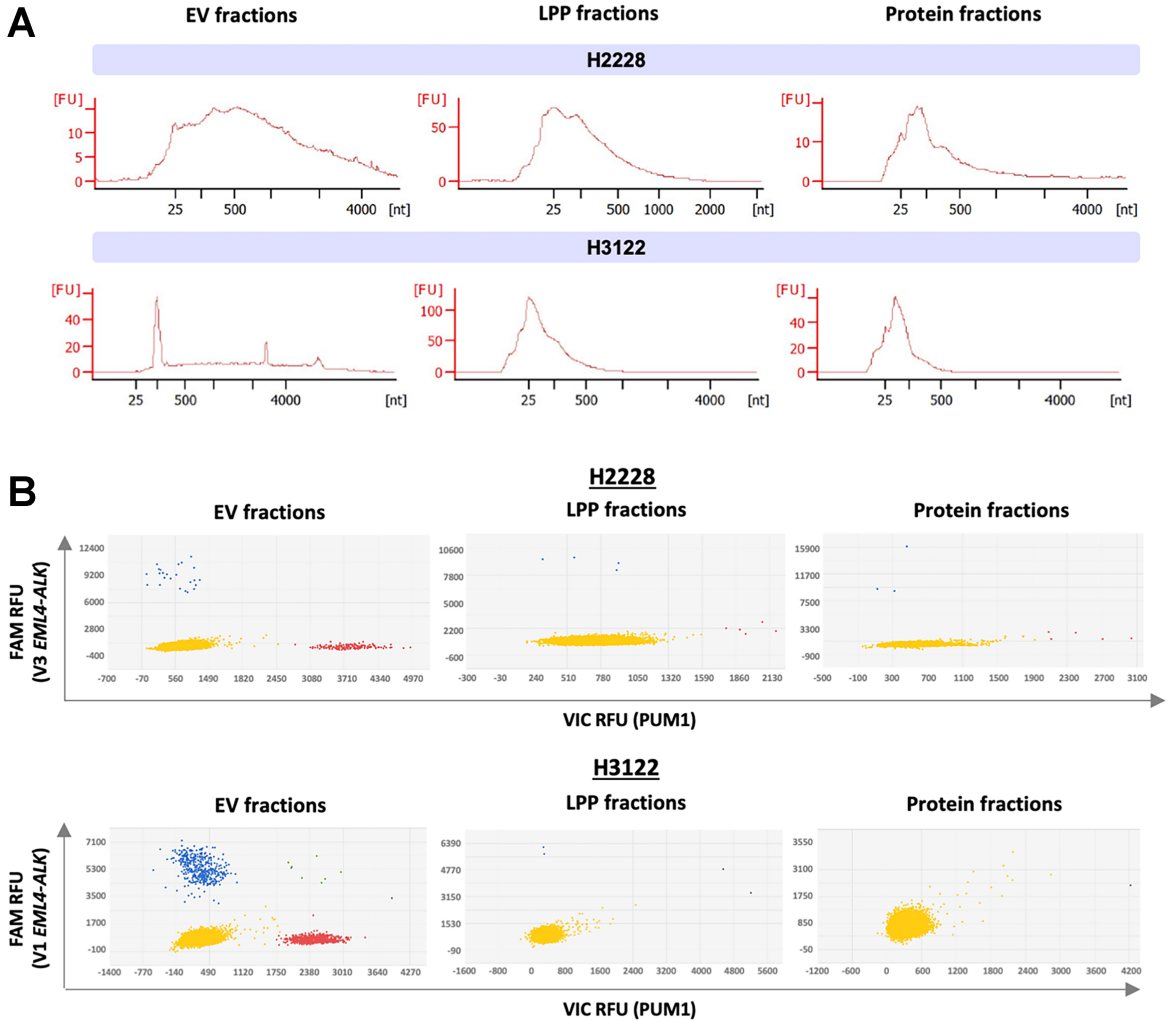
Analysis of *EML4-ALK* fusion transcripts in SEC fractions from H2228 and H3122 cell lines. SEC fractions 9-11 (EVs), 15-17 (LPPs), and 19-21 (protein) for H2228, and fractions 9-12 (EVs), 14-16 (LPPs), and 19-21 (protein) for H3122, were pooled and subjected to RNA extraction. (A) RNA size profiles in pooled fractions, analyzed using the Bioanalyzer RNA 6000 Pico assay. Graphing software: Agilent 2100 Expert Software; (B) Detection of *EML4-ALK* translocation in EV-, LPP-, or protein-enriched fractions using dPCR. A variant 3 TaqMan assay was used for H2228 samples, and a variant 1 assay for H3122. Graphing software: QuantStudio 3D AnalysisSuite Cloud Software. Each dot represents a well on the dPCR chip. Blue dots indicate wells with at least one copy of the *EML4-ALK* translocation (FAM); red dots represent the detection of the endogenous *PUM1* gene (VIC); Yellow and green dots indicate wells negative or positive for both genes, respectively; Grey dots represent wells not classified by the analysis software. EML4-ALK: Echinoderm microtubule-associated protein-like 4-anaplastic lymphoma kinase; SEC: size-exclusion chromatography; EV: extracellular vesicle; LPP: lipoprotein particle; dPCR: digital polymerase chain reaction; RNA: ribonucleic acid; FAM: 6-carboxyfluorescein; PUM1: pumilio RNA binding family member 1.

Subsequently, RNA was extracted from the various pooled fractions using the Norgen Plasma/Serum Exosome and Free-Circulating RNA Isolation kit. RNA was detected in all fractions [Figure 2A], and the presence of the *EML4-ALK* translocation in each population was analyzed by dPCR. In digital PCR, the sample is distributed in one chip with thousands of wells, so ideally, only one RNA molecule of interest will be present in each well. An individualized PCR reaction is performed in each well, containing primers designed to amplify two different sequences: the target sequence and a control sequence (either mRNA from a specific *EML4-ALK* translocation variant or from the *PUM1* gene, respectively). If either of the target sequences is amplified, an increase in fluorescence can be detected using FAM and VIC fluorophore probes, respectively. The analysis software then assigns the wells to different groups on the graph: yellow dots represent wells with no amplification of either of the two target sequences; red dots indicate amplification of PUM1; blue dots correspond to amplification of the specific EML4-ALK variant; and green dots indicate amplification of both genes. dPCR analysis revealed that the endogenous control *PUM1* and the translocation transcripts were enriched in EV fractions compared to LPPs or free protein in both cell lines [Figure 2B]. In EVs, an average of 6.5 and 92 copies of the fusion transcript per mL of CM was detected, with mutant allele frequencies of 21.4% and 32% for the H2228 and H3122 lines, respectively.

### Optimization of RNA extraction for the detection of transcripts of endogenous *PUM1* and *EML4-ALK* translocation

Previous studies detecting *ALK* translocations in patients’ plasma used large sample volumes^[19,20]^, which would further increase after SEC and lysis. Processing such large volumes delays RNA binding to the isolation column, as several centrifugation steps are required to pass the entire sample volume through. Since this step is performed at RT, it may lead to a significant degradation of the genetic material. To address this, we optimized the RNA extraction protocol by testing Qiagen’s “RNeasy MinElute Spin Columns”, which are compatible with a vacuum system. This setup reduces RNA binding time following TRIzol/chloroform extraction.

**Figure 3:**
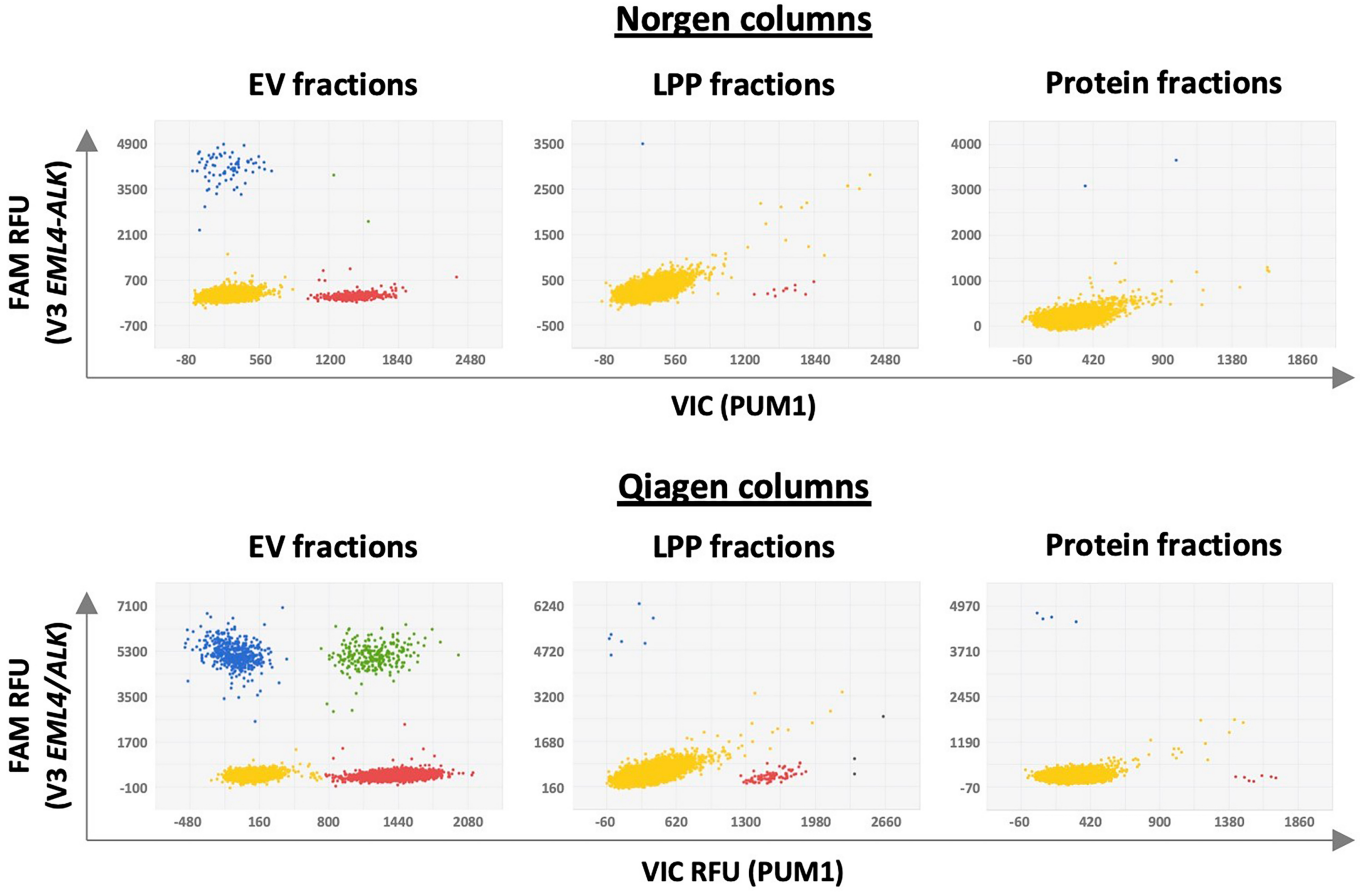
Comparison of two RNA extraction columns (Norgen *vs*. Qiagen) for detecting the *EML4-ALK* translocation by dPCR. EV-enriched (fractions 9-11), LPP-enriched (15-17), and protein-enriched (19-21) fractions from H2228 were used. RNA was eluted in 40 µL (Norgen) or 14 µL (Qiagen). From each, 6.5 µL was used for cDNA synthesis. Each dot represents a well on the dPCR chip. Blue dots represent wells with at least one copy of the EML4-ALK translocation, (FAM fluorophore); red dots show detection of the endogenous *PUM1* gene (VIC fluorophore). Yellow and green dots indicate wells negative or positive for both genes, respectively. Grey dots represent wells not assigned by the analysis software. Graphing software: QuantStudio 3D AnalysisSuite Cloud Software. EML4-ALK: Echinoderm microtubule-associated protein-like 4-anaplastic lymphoma kinase; cDNA: complementary DNA; dPCR: digital polymerase chain reaction; EV: extracellular vesicle; FAM: 6-carboxyfluorescein; LPP: large protein particle; PUM1: pumilio RNA-binding family member 1; RNA: ribonucleic acid.

These modifications were first tested on EV-, LPP-, and free protein-enriched fractions obtained from concentrated and SEC-separated CM from the H2228 cell line. RNA was extracted from each of the three components using TRIzol/chloroform, followed by isolation from the aqueous phase using either Norgen Mini Spin Columns or Qiagen RNeasy MinElute Spin Columns with a vacuum system. After washing, RNA was eluted in 40 µL (Norgen) or 14 µL (Qiagen). The RNA concentrations were 7.5 and 7.42 ng/µL for EVs and soluble proteins isolated with Qiagen columns, respectively, while concentrations in the other samples were below the detection limit of the Qubit kit. The use of vacuum-assisted columns clearly improved RNA isolation, enabling the detection of up to 10 times more copies of the EML4-ALK translocation and 20 times more *PUM1* transcripts [Figure 3].

### Detection of transcripts for *EML4-ALK* translocation in pooled plasma from ALK-positive NSCLC

**Figure 4:**
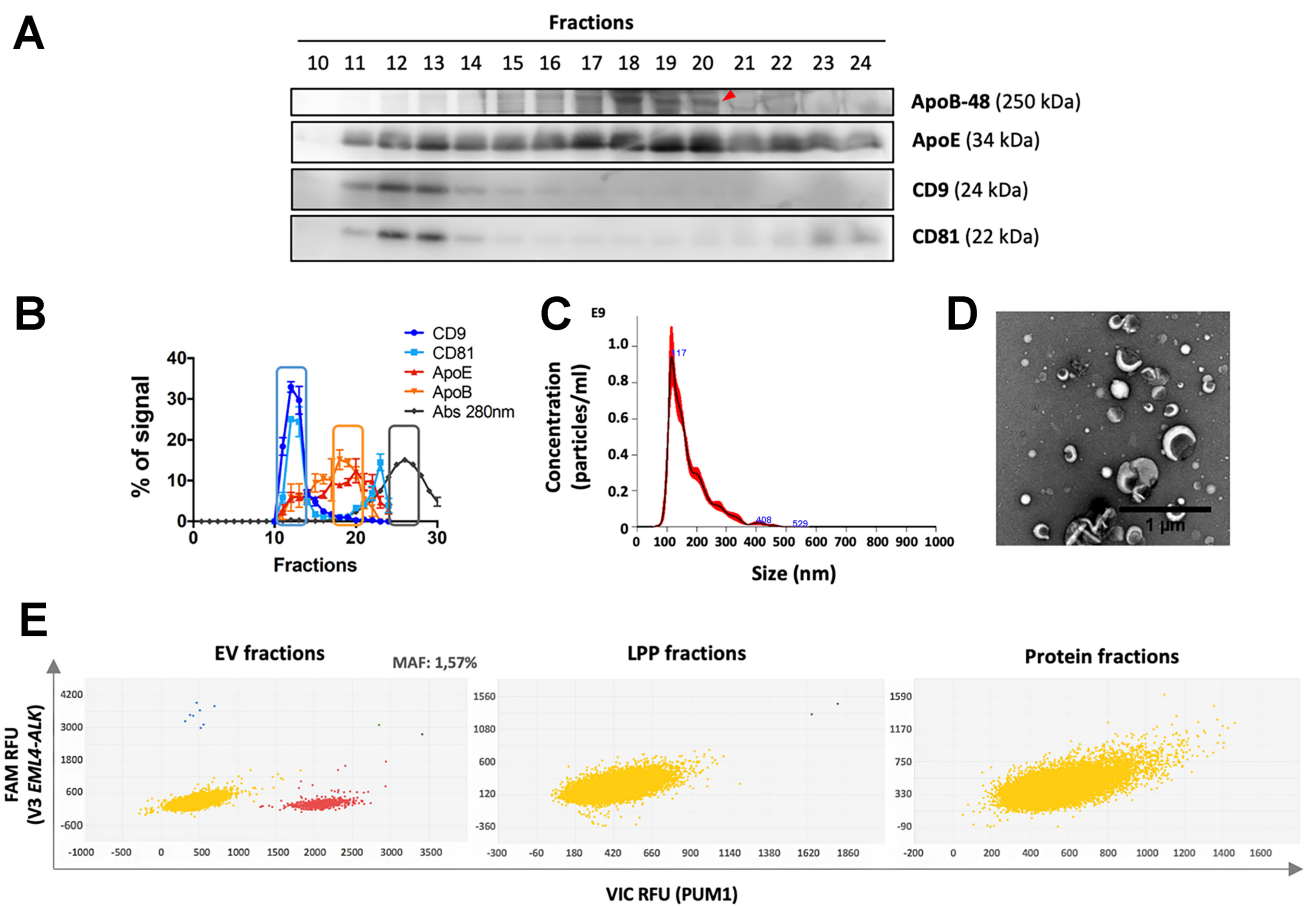
Detection of *EML4-ALK* translocation in plasma samples from *EML4-ALK*-positive NSCLC patients. EVs, LPPs, and soluble proteins were separated using a 13 mL SEC column packed with 4RRF resin. (A) Representative western blot detecting EV markers (CD81) and LPP markers [ApoE and ApoB (indicated by a red arrow)]. 20 μL of each SEC fraction were loaded per well. Graphing software: LAS 4000 mini system and Fiji ImageJ; (B) Elution profiles of EVs, LPPs, and soluble proteins. Densitometry analysis of western blots for EV markers (CD9 and CD81) and LPP markers (ApoE and ApoB), alongside absorbance at 280 nm for total protein. The % signal for each marker in each fraction is shown (100% represents the sum of signals for that marker across all eluted fractions). Squares indicate pooled fractions for further analysis. Data represent mean ± SEM of four independent runs. Graphing software: GraphPad Prism; (C) Particle size distribution in EV-enriched fractions, measured by NTA. Graphing software: NTA 3.0 software; (D) Representative TEM images of EV-enriched fractions after negative staining. Scale bars = 1 μm. Graphing software: JEM1400 Flash transmission electron microscope (Jeol) and Image J; (E) Detection of EML4-ALK translocation by dPCR in EV-enriched (fractions 11-13), LPP-enriched (18-20), and protein-enriched (25-27) fractions. Graphing software: QuantStudio 3D AnalysisSuite Cloud Software. Each dot represents a well on the dPCR chip. Blue dots represent wells with at least one copy of the *EML4-ALK* translocation (FAM fluorophore); red dots show the detection of the endogenous *PUM1* gene (VIC fluorophore); Yellow and green dots indicate wells negative or positive for both targets, respectively. ALK: Anaplastic lymphoma kinase; dPCR: digital polymerase chain reaction; EML4: echinoderm microtubule-associated protein-like 4; EVs: extracellular vesicles; FAM: 6-carboxyfluorescein; LPPs: lipoprotein particles; NTA: nanoparticle tracking analysis; NSCLC: non-small cell lung cancer; PUM1: pumilio homolog 1; SEC: size-exclusion chromatography; SEM: standard error of the mean; TEM: transmission electron microscopy.

To adapt the SEC protocol for use with plasma samples, which are more viscous than conditioned media, we directly loaded 1 mL of plasma per column onto taller 13 mL columns packed with 4RRF resin, omitting the preconcentration step. As shown by Western blot analysis and the sample’s elution profile [Figure 4A and B], the resolution between EVs, LPPs, and soluble proteins was satisfactory. EV-enriched fractions were further characterized by NTA [Figure 4C], which revealed a size distribution centered around 110 nm in diameter, and by TEM [Figure 4D] to confirm proper EV isolation. Zeta potential measurements yielded values of -10.37 ± 0.56 mV for EV fractions and -7.08 ± 1.06 mV for LPP-enriched fractions. After the dPCR reaction, we detected predominant levels of *PUM1* and the *EML4-ALK* translocation in EV-enriched fractions, but not in LPP or protein fractions, confirming that RNA is preferentially transported in EVs [Figure 4E]. Specifically, we identified 4.56 *EML4-ALK* fusion copies/EVs in 1 mL of plasma, corresponding to a MAF of 1.57%.

## DISCUSSION

Detecting complex genomic alterations such as translocations through liquid biopsy remains challenging. In this regard, the use of EVs is promising^[28]^, although other particles such as LPPs^[13-15]^ and certain protein complexes have also been shown to transport RNA molecules^[16]^. Previous studies have detected *ALK* translocations in EV-enriched samples^[19,20]^; however, the EV isolation methods used in those studies may have led to the co-isolation of protein precipitates or other contaminants. Here, we aimed to directly study the presence of *EML4-ALK* translocation mRNA not only in EVs but also in fractions enriched in LPPs and free proteins. To this end, we isolated EVs using SEC with an optimized resin, which, according to our previous data, enables improved separation of the three main components (EVs, LPPs, and proteins) from both conditioned media and plasma samples^[23]^.

First, we demonstrated that the *EML4-ALK* translocation was primarily detected in purified EVs derived from the secretome of NSCLC cell lines carrying the two most common variants of this fusion (H3122, variant 1; H2228, variant 3). A few copies were also detected in LPP- or protein-enriched fractions in some replicates, possibly originating from smaller EVs that elute later into these fractions. Moreover, the endogenous *PUM1* transcript was predominantly found in the EV fraction, further supporting the idea that most secreted transcripts are encapsulated in EVs, likely due to either preferential secretion through this pathway or improved RNA preservation in the extracellular environment.

The SEC resin used in our study [4% Rapid Run Agarose Bead Fine, ABT (4RRF)] has an approximate exclusion limit of 40 nm. Consequently, particles larger than 40 nm, including the vast majority of EVs, were collected in the EV fraction. Interestingly, the H3122 cell line seems to secrete EVs of more heterogeneous sizes than H2228, especially smaller EVs. This is evident from the elution profile of EV markers analyzed by dot blot, which shows a secondary EV peak in the LPP-enriched fractions. Despite the presence of small EVs in the LPP fractions of H3122-derived samples, we did not observe a corresponding increase in *PUM1* or *EML4-ALK* transcript levels, suggesting that these very small EVs carry minimal amounts of mRNAs. This may be due to reduced vesicle volume or selective incorporation of mRNAs into larger EVs. Similar to miRNAs^[29]^, mRNA loading into EVs is a selective process, as the transcriptome of EVs often differs from that of the parent cells. In fact, previous studies have shown greater transcriptomic similarity between cells and microvesicles than between cells and exosomes^[30]^. Specific sequences have also been proposed to mediate the selective incorporation of certain miRNAs and mRNAs into EVs^[31]^.

We next investigated whether the *EML4-ALK* translocation is similarly enriched in EVs derived from plasma. A key challenge in this context is that SEC-based EV isolation results in significant sample dilution, requiring the processing of large volumes and prolonged RNA-binding times, which in turn reduce RNA purification efficiency. We resolved this issue by applying vacuum during RNA extraction, which accelerated RNA binding to the columns. We also observed that the mini Norgen column became saturated with the sample volume used, whereas the Qiagen column exhibited higher binding capacity or was less affected by prolonged processing times.

To simplify the procedure and to avoid increasing the isolated sample volume, we eliminated the preconcentration step and employed taller SEC columns (13 mL packed resin), enabling isolation from 1 mL of plasma while still achieving good resolution of sample components. With these optimizations, we successfully detected the *EML4-ALK* translocation by dPCR exclusively in EV-enriched fractions from 8 mL of plasma, with no signal detected in LPP- or protein-enriched fractions. This confirms the specific association of the *EML4-ALK* transcript with EVs.

ALK-positive NSCLC is relatively rare, accounting for approximately 5% of all metastatic NSCLC cases. Consequently, patient recruitment for studies focused on this subgroup is challenging and often results in small sample sizes. Nevertheless, our findings remain clinically meaningful given the markedly improved survival outcomes in this patient population. For example, data from the CROWN trial showed that approximately 90% of ALK-positive NSCLC patients treated with lorlatinib were alive at 12 months, compared to only about 40% of patients in the broader metastatic NSCLC population^[8]^. This underscores the importance of biomarker testing in guiding treatment decisions.

Our study thus provides both a proof of concept and a practical demonstration of the feasibility - as well as the technical challenges - of detecting *EML4-ALK* fusion transcripts in patient plasma. Current liquid biopsy methods for detecting ALK translocations, which are typically identified via tissue biopsy, lack adequate sensitivity^[32]^, potentially compromising treatment decisions and negatively affecting survival. In this study, we show that ALK mRNA is specifically enriched in the vesicular fraction of plasma, indicating that improving EV isolation techniques will be essential for clinical translation. Enhancing the efficiency of EV recovery could reduce false-negative results and strengthen the role of liquid biopsy in treatment planning. Notably, other actionable fusions, such as NTRK^[33]^, ROS1^[34]^, and RET, also identify patients eligible for targeted therapies.

### Conclusion

In summary, our data demonstrate that the ALK fusion oncogenic driver is exclusively secreted via EVs. This finding highlights the potential for future developments aimed at leveraging this feature to facilitate biomarker testing through EVs. Furthermore, our results support the hypothesis that cancer may not only be an evolutionary process but could also be viewed as an infectious disease. This perspective may open new avenues for the development of treatment strategies.

## DECLARATIONS

### Availability of data and materials

Raw data and materials are available from the corresponding authors upon reasonable request.

### Copyright

© The Author(s) 2025.
