# Supplementary material for: Optimized size exclusion chromatography demonstrates that extracellular vesicles are the key RNA carriers of ALK translocations in non-small cell lung cancer cell line secretome and patient plasma
Source: Extracell Vesicles Circ Nucl Acids. 2025 Jun 18;6(2):310–23. doi: 10.20517/evcna.2025.14 (PMC12367459; doi:10.20517/evcna.2025.14)
Supplement: Supplementary file 1 [file evcna-6-2-310-SupplementaryMaterials.pdf]

Supplementary Materials

Optimized size exclusion chromatography demonstrates that extracellular vesicles are the key RNA carriers of ALK translocations in non-small cell lung cancer cell line secretome and patient plasma

Beatriz Benayas[1,#], Estela Sánchez-Herrero[2,#], Lucía Robado de Lope[3,#], Joaquín Morales[1], Soraya López-Martín[1], Mariano Provencio[3], Mar Valés-Gómez[2], Atocha Romero[3], María Yáñez-Mó[1]

[1]Centro de Biología Molecular Severo Ochoa (CBM), IIS-Princesa, Universidad Autónoma de Madrid, IUBM, Madrid 28049, Spain.
[2]Immunology and Oncology Department, Spanish National Centre for Biotechnology (CNB-CSIC), Madrid 28049, Spain.
[3]Liquid Biopsy Laboratory, Medical Oncology Department, Hospital Puerta de Hierro, IDHIPISA, Madrid 28022, Spain.
[#]Authors contributed equally.

Correspondence to: Dr. Mar Valés-Gómez, Immunology and Oncology Department, Spanish National Centre for Biotechnology (CNB-CSIC), Lab 417, C/ Darwin, 3, Madrid 28049, Spain. E-mail: mvales@cnb.csic.es; Dr. Atocha Romero, Liquid Biopsy Laboratory, Medical Oncology Department, Hospital Puerta de Hierro, IDHIPISA, C/ Manuel de Falla 1, Madrid 28022, Spain. E-mail: Atocha10@hotmail.com; Dr. María Yáñez-Mó, Centro de Biología Molecular Severo Ochoa (CBM), IIS-Princesa, Universidad Autónoma de Madrid, IUBM, Lab 412, C/ Nicolás Cabrera, 1, Madrid 28049, Spain. E-mail: maria.yannez@uam.es

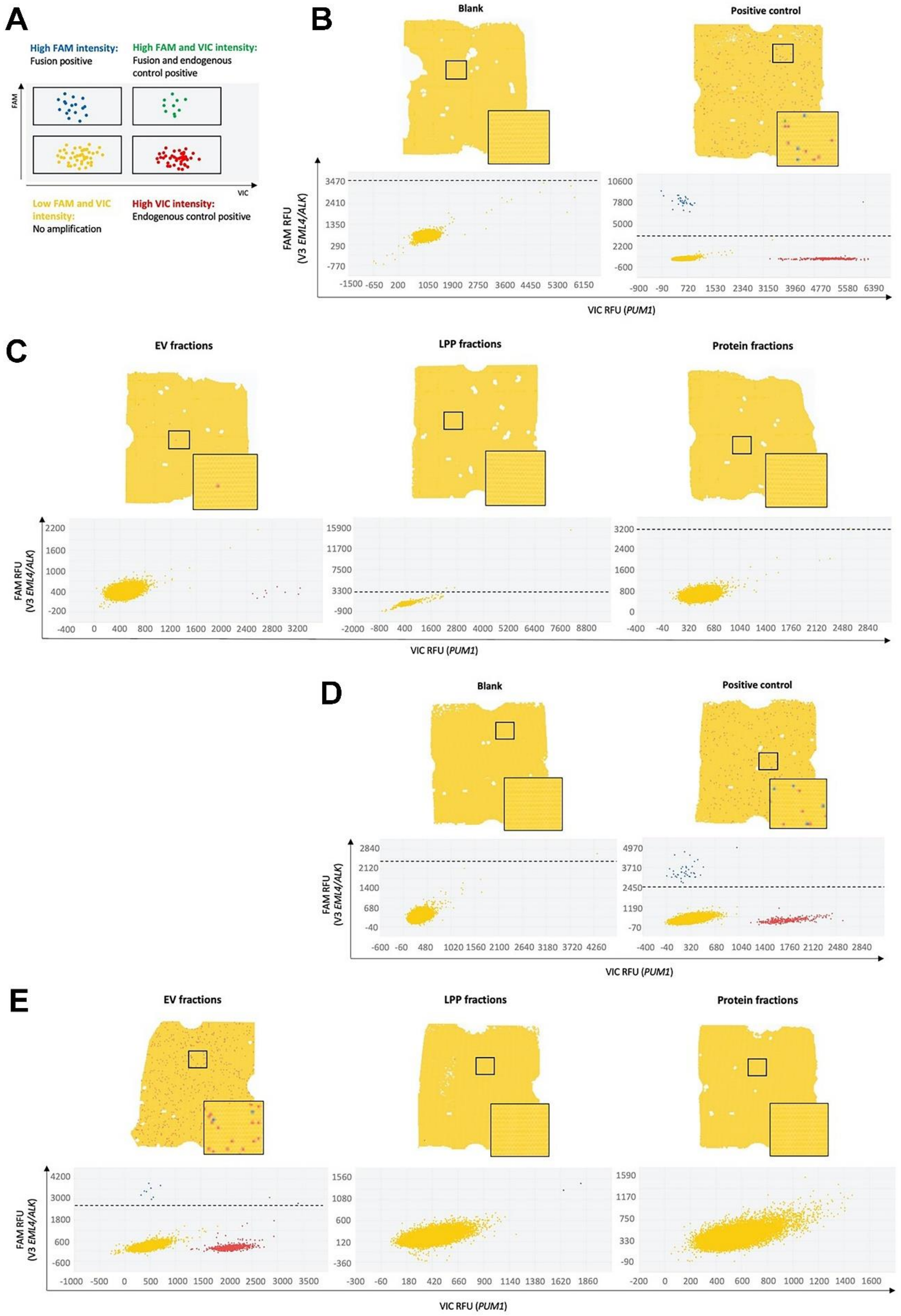

Supplementary Figure 1. Digital PCR Analysis. The presence of *EML4-ALK* translocation was checked by digital PCR (dPCR) using the Digital QuantStudio® 3D Digital PCR 20K chip. The dPCR reaction mixture included TaqMan oligo to detect

PUM1 (Dye VIC-MGB) as an endogenous gene, and TaqMan oligos EML4(13):ALK(20) or TaqMan EML4(6a/b):ALK(20) (Dye FAM-MGB) to detect variants 1 and 3 of the translocation, respectively. Each dPCR included a blank (no cDNA), and a positive control (cell lysate from H2228 or H3122) (B and D). With these controls, thresholds of positive signal are determined and applied to SEC fractions from negative (C) and ALK carrying plasma samples (E). After dPCR reaction, the chips were read twice. The figure depicts the image of each chip together with the plot of the results obtained upon analysis with the QuantStudio® 3D AnalysisSuite™ Cloud program that categorizes each spot into the four main components as in (A).
